# Exposure–response analysis of alectinib in crizotinib-resistant ALK-positive non-small cell lung cancer

**DOI:** 10.1007/s00280-018-3597-5

**Published:** 2018-05-10

**Authors:** Peter N. Morcos, Eveline Nueesch, Felix Jaminion, Elena Guerini, Joy C. Hsu, Walter Bordogna, Bogdana Balas, Francois Mercier

**Affiliations:** 1 Roche Innovation Center New York, New York, NY USA; 2 F. Hoffmann-La Roche Ltd, Basel, Switzerland; 3 Roche Innovation Center Basel, Basel, Switzerland

**Keywords:** Alectinib, ALK inhibitor, NSCLC, Exposure response, ER, Pharmacokinetics, Oncology

## Abstract

**Purpose:**

Alectinib is a selective and potent anaplastic lymphoma kinase (ALK) inhibitor that is active in the central nervous system (CNS). Alectinib demonstrated robust efficacy in a pooled analysis of two single-arm, open-label phase II studies (NP28673, NCT01801111; NP28761, NCT01871805) in crizotinib-resistant *ALK*-positive non-small-cell lung cancer (NSCLC): median overall survival (OS) 29.1 months (95% confidence interval [CI]: 21.3–39.0) for alectinib 600 mg twice daily (BID). We investigated exposure–response relationships from final pooled phase II OS and safety data to assess alectinib dose selection.

**Methods:**

A semi-parametric Cox proportional hazards model analyzed relationships between individual median observed steady-state trough concentrations (*C*_trough,ss_) for combined exposure of alectinib and its major metabolite (M4), baseline covariates (demographics and disease characteristics) and OS. Univariate logistic regression analysis analyzed relationships between *C*_trough,ss_ and incidence of adverse events (AEs: serious and Grade ≥ 3).

**Results:**

Overall, 92% of patients (*n* = 207/225) had *C*_trough,ss_ data and were included in the analysis. No statistically significant relationship was found between *C*_trough,ss_ and OS following alectinib treatment. The only baseline covariates that statistically influenced OS were baseline tumor size and prior crizotinib treatment duration. Larger baseline tumor size and shorter prior crizotinib treatment were both associated with shorter OS. Logistic regression confirmed no significant relationship between *C*_trough,ss_ and AEs.

**Conclusion:**

Alectinib 600 mg BID provides systemic exposures at plateau of response for OS while maintaining a well-tolerated safety profile. This analysis confirms alectinib 600 mg BID as the recommended global dose for patients with crizotinib-resistant *ALK*-positive NSCLC.

**Electronic supplementary material:**

The online version of this article (10.1007/s00280-018-3597-5) contains supplementary material, which is available to authorized users.

## Introduction

Rearrangement of the anaplastic lymphoma kinase (*ALK*) gene occurs in approximately 5% of patients with advanced non-small-cell lung cancer (*ALK*-positive NSCLC) [1]. This disease is characterized by high risk of central nervous system (CNS) metastases [2], and a high prevalence of brain metastases at diagnosis [3, 4]. Crizotinib was the historic standard of care for first-line treatment of *ALK*-positive NSCLC, based on improved progression-free survival (PFS) compared with standard chemotherapy (10.9 months versus 7.7 months, respectively) in the phase III PROFILE 1014 trial [5]. However, many patients treated with crizotinib relapse within 1 year, primarily due to poor CNS penetration or development of resistance mutations [6, 7]. As such, there was an unmet medical need to overcome the challenges associated with crizotinib treatment.

Alectinib is a potent ALK tyrosine kinase inhibitor (TKI) with an IC_50_ (50% of maximum inhibitory concentration) of 1.9 nmol/L in enzymatic analyses [8]. Alectinib has demonstrated antitumor activity in preclinical models that are resistant to the previous standard of care, crizotinib, as well as CNS tumor models [9, 10]. Two single-arm, phase II studies (NP28673, global [NCT01801111] [11] and NP28761, North American [NCT01871805] [12]) demonstrated robust efficacy and an acceptable safety profile for alectinib 600 mg twice daily (BID) in patients with *ALK*-positive NSCLC following crizotinib failure. An updated pooled analysis for these studies at a median follow-up of 92.29 weeks demonstrated a median overall survival (OS) of 29.1 months (95% confidence interval [CI]: 21.3–39.0), and good tolerability over a median treatment duration of 11.3 months with alectinib [13]. The pivotal global, randomized, phase III ALEX study (BO28984, NCT02075840) confirmed the clinical benefit of first-line alectinib treatment for patients with ALK inhibitor naïve *ALK*-positive NSCLC [14]. In ALEX, the primary endpoint (investigator-assessed median PFS) was significantly improved with alectinib versus crizotinib: hazard ratio (HR) for disease progression or death was 0.47 (95% CI: 0.34–0.65; *P* < 0.001) [14]. Based on these cumulative data, the globally approved dosing regimen for alectinib is 600 mg BID.

Dose selection is a critical issue for development and registration of new molecular entities (NMEs). Optimal dose selection is achieved by maximizing the potential for efficacy, while minimizing the potential for safety risks in the target patient population, and is a key review issue by major health authorities during new drug application (NDA) reviews [15]. Identification of optimal dose selection is a challenging task during drug development, particularly in the field of oncology, where first-in-human studies primarily use conventional approaches originally tailored for cytotoxic chemotherapeutics and subsequent development programs focus on speed to approval [16–18]. The challenges associated with optimizing dose selection for oncology NMEs is highlighted in recent reviews of regulatory evaluations of NDAs [16, 19]. Indeed, in one review it is cited that more than one quarter of approved new oncology agents within an investigated period required further dose optimization investigations, with a greater trend reported for approved TKIs [19]. Thus, there is regulatory expectation to maximize the benefit/risk of the NME for approval and use in the intended patient population by justifying or investigating optimal dose selection.

To robustly justify dose selection for an NME, the cumulative available nonclinical and clinical data are generally considered to ensure its optimal use. A clinical evaluation of dose selection is based on the established efficacy and safety along with a thorough characterization of exposure–response relationships for those established efficacy and safety data. The following analyses examine the exposure–response relationships for alectinib in the crizotinib-resistant *ALK*-positive NSCLC population based on final pooled efficacy and safety data from the pivotal phase II North American study (NP28761; NCT01871805) and the phase II global study (NP28673; NCT01801111) to assess alectinib dose selection in this setting.

## Materials and methods

### Study design and patient population

The study designs for the two studies have been published previously [11, 12]. In brief, both were single-arm, open-label, multicenter studies that enrolled patients with *ALK*-rearranged, locally advanced or metastatic NSCLC, who experienced progression on, or were intolerant to, crizotinib therapy, with a primary objective to assess efficacy and safety. Inclusion criteria for both studies were: histologically confirmed, advanced *ALK*-rearranged NSCLC (as assessed by an FDA-approved test), progression on crizotinib treatment, aged ≥ 18 years with Eastern Cooperative Oncology Group performance status ≤ 2, adequate organ function, and measurable disease. Patients with stable treated brain and/or leptomeningeal metastases or asymptomatic untreated brain and/or leptomeningeal metastases were eligible for enrolment. A minimum 7-day wash-out period was required between the last crizotinib dose and the first alectinib dose [11, 12]. In both studies, patients received 600 mg oral alectinib administered with food BID until progression, death, or withdrawal. Both studies were conducted in accordance with the Declaration of Helsinki and Good Clinical Practice guidelines and all patients provided written informed consent.

### Analysis data

The efficacy analysis dataset consisted of the final pooled OS data from the two individual single-arm phase II trials [13]. The pooled analysis assessed OS and safety after median follow-up of 92.29 weeks (NP28673: 105.5 weeks, data cut-off 27 October 2017; NP28761: 75.71 weeks, data cut-off 12 October 2017). The safety analysis dataset focused on 2 key safety events generally associated with overall tolerability of study drug: any serious adverse event (SAE) and any Grade ≥ 3 AE reported by patients during the study and throughout the follow-up period. Adverse events (AEs) were coded using Medical Dictionary for Regulatory Activities version 17.1; severity of AEs was coded according to the Common Terminology Criteria for Adverse Events version 4.0.

In both studies, approximately 2 mL of venous blood was collected in all patients for pharmacokinetic (PK) analysis of alectinib and its major active metabolite, M4, at various timepoints throughout the study periods [11, 12]. Alectinib and M4 plasma concentrations were determined simultaneously using validated liquid chromatography tandem mass spectroscopy using a separate laboratory for each study. Cross-validation experiments revealed an analytical bias of approximately 20% between the two laboratories and, therefore, concentrations of alectinib and M4 were adjusted to account for the analytical bias. Full details on the bioanalytical methodology for alectinib and M4 including cross-validation experiment results are presented elsewhere [20]. For both alectinib and M4, the observed steady-state trough concentration, defined as the median value of all available pre-dose concentrations in each patient collected after Day 8 (steady-state) of dosing was used as the exposure measure for exposure–response analyses. Patient samples were excluded if they were taken after drug administration or collected within 8 days of a documented dose deviation during the study. Both alectinib and its major metabolite, M4, have demonstrated similar in vitro potency and activity against the target ALK kinase and similar protein binding supporting that both analytes contribute to overall alectinib efficacy and safety [21]. Therefore, the combined exposure of alectinib and M4 (alectinib + M4) steady-state trough concentration adjusted for the individual molecular weights was utilized as the overall alectinib exposure measure (herein *C*_trough,ss_) in the exposure–response analyses. This approach has been utilized for all other alectinib exposure investigations [22–25] and similarly utilized for exposure–response assessments for other agents with active metabolites [26–28].

### Exposure-efficacy analysis

The primary efficacy measure explored in the exposure-efficacy analysis was OS reported from the final datacut of the pooled phase II analyses in the crizotinib-resistant patient population [13]. The relationship between alectinib exposure and OS was investigated using a semi-parametric Cox proportional hazards (CPH) model. The statistical methodology underpinning the analysis technique has been described in detail in a seminal paper [29]. Briefly, the survival analysis technique describes the relationship between the distribution of survival and pre-specified covariates [30]. A multiplicative model of the hazard could be described as: *h*_*i*_(*t*) = *h*_*0*_(*t*) *exp*(*β*_*1*_*x*_*i1*_ + *β*_*2*_*x*_*i2*_ + · · · + *β*_*k*_*x*_*ik*_). Here, *h*_*0*_(*t*) is the baseline hazard function, *X*_*i*_ = {*x*_*i1*_, *x*_*i2*_,…, *x*_*ik*_} is the vector of covariates for individual *i*, and *β*_*k*_ is the coefficient which corresponds to covariate *k*. The model assumes a baseline hazard which is common to all individuals included in the study population. The covariates have multiplicative effect on the baseline hazard. Further details can be found elsewhere [29, 30].

The investigation of the relationship between alectinib exposure along with baseline covariates and OS was conducted in two steps. First, a base model investigated the effect of alectinib exposure on OS without consideration of any potential effects of covariates. Several functional forms of alectinib exposure were considered to thoroughly investigate the potential exposure relationship to OS: linear continuous variables of exposure (*C*_trough,ss_ and the logarithm of *C*_trough,ss_ [i.e., log(*C*_trough,ss_)]), linear categorical forms (halves and tertiles), and nonlinear forms (*E*_max_-type function) were all investigated in the base model. The objective function value (*OF*, corresponding to *−* 2 *x* log-partial likelihood) and the Bayesian Information Criterion (BIC) were used to select the best base model. BIC was computed as *OF* + *p* × log(*n*), where *n* is the number of observations and *p* is the number of model parameters. The model with the lowest BIC value was selected as the base model.

In the second step, covariates were investigated on the base model. Initially, a univariate screening was undertaken where every covariate was added one at a time to the base model. Covariates leading to a lower BIC values than the one observed with the base model were carried forward to the next model building step. The selected covariates were sequentially added (forward inclusion) to the base model and retained in a full covariate model if it further reduced the BIC value. After the full covariate model was developed, a backward elimination process was finally performed where covariates were excluded from the full model one at a time, and BIC values of the reduced and the full models were compared. The covariate was eliminated if the BIC value of the reduced model was lower than that of the full model. The final model, therefore, retained only the significant covariates influencing alectinib OS.

The adequacy of the final fitted CPH model was confirmed through creation of diagnostic plots. Schoenfeld residual plots were used to check the assumption of proportional hazards and examined whether there was any marked time-pattern in the residuals. Martingale residual plots were used to investigate any nonlinearity (i.e., an incorrectly specified functional form in the model) for each included covariate in the final model. Finally, a visual assessment of the performance of the final model was obtained by overlaying of the observed OS from the nonparametric Kaplan–Meier analysis of the pooled phase II studies to the final CPH model and associated 95% CIs.

### Exposure-safety analysis

The relationship between alectinib exposure and the investigated safety endpoints, SAEs and Grade ≥ 3 AEs, was investigated through univariate logistic regression analyses. A Chi-square statistic was used for assessment of statistical significance of the relationship. In addition, the distribution of individual grades of AEs was assessed by graphically presenting the distribution of alectinib exposure by each grade of event for SAEs and Grade ≥ 3 AEs. An exploratory analysis of variance (ANOVA) was used for assessment of statistical significance in the distributions by AE grades.

The analysis and all figures were conducted and produced using R (Version 3.2.4) [31].

## Results

### Population

**Fig. 1:**
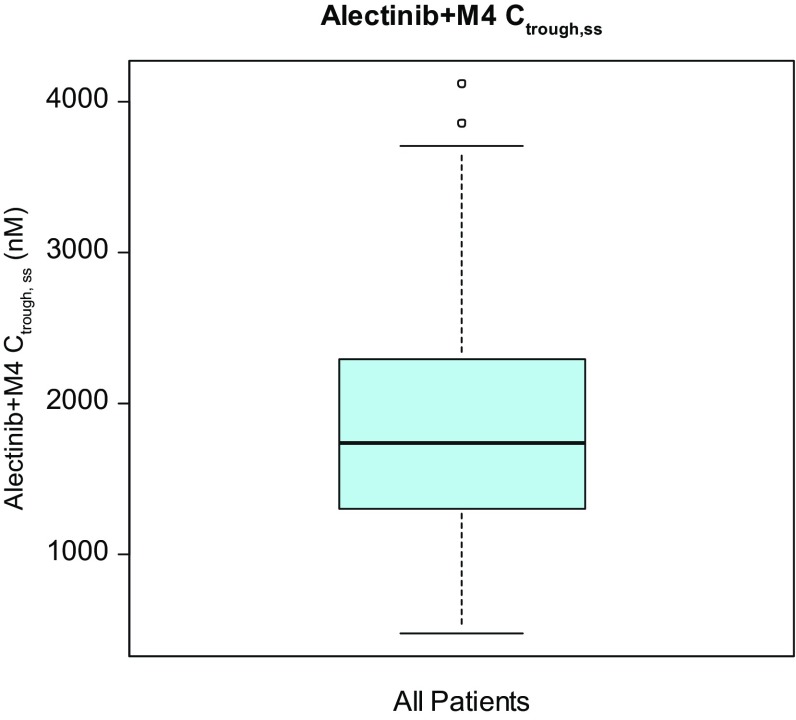
Distribution of alectinib + M4 *C*_trough,ss_ across all patients in exposure–response population. The black line in the center of the box represents the median value of the continuous *C*_trough,ss_. Boxes indicate the inter-quartile range (IQR). Whiskers represent 1.5*IQR. Outliers are marked outside of the whiskers by circles

A total of 225 patients were enrolled across both studies and received alectinib 600 mg BID [13]. The exposure–response population included 207 (92%) of these patients with available *C*_trough,ss_ data. Baseline characteristics included in the analysis population are provided in Table 1. Overall the baseline factors from these patients were representative of the expected *ALK*-positive NSCLC population [31] and consistent with the overall population from the pooled phase II studies [13]. There were slightly more females than males and patients were generally young with a mean age of 52.3 years in the exposure–response population. The distribution of alectinib exposure (C_trough,ss_) encompasses nearly a ninefold exposure range supporting a robust exploration of exposure–response relationships across wide exposures (Fig. 1).

**Table 1:** Summary of baseline covariates in the exposure–response population

Covariate	*n* = 207
Race, *n* (%)	
White	151 (72.9)
Asian	42 (20.3)
Other	14 (6.8)
Ethnicity, *n* (%)	
Hispanic	17 (8.0)
Non-hispanic	190 (92.0)
Sex, *n* (%)	
Female	112 (54.0)
Male	95 (46.0)
Age (years), mean (SD)	52.3 (11.4)
Body weight (kg), mean (SD)	73.5 (17.7)
Height (cm), mean (SD)	168.1 (10.3)
BMI (Kg/m^2^), mean (SD)	25.9 (5.4)
BSA (m^2^), mean (SD)	1.8 (0.2)
Baseline tumor size (mm), mean (SD)	56.2 (45.0)
Smoking status at baseline, *n* (%)	
Non-smoker	143 (69.1)
Past or present smoker	64 (30.9)
CNS metastases at baseline, *n* (%)	
Yes	48 (23.0)
No	159 (77.0)
Prior chemotherapy status, *n* (%)	
Yes	158 (76.0)
No	49 (24.0)
Prior crizotinib treatment duration (days), mean (SD)	438 (293.2)
Category of ECOG score at baseline, *n* (%)	
ECOG score 0 or 1	185 (89.0)
ECOG score 2	22 (11.0)
Alectinib + M4 *C*_trough,ss_, Geo Mean (Geo Mean CV%)	1680 (45.2)

*BMI* Body mass index, *BSA* body surface area, *ECOG* Eastern Cooperative Oncology Group, *Geo mean* Geometric mean, *SD* standard deviation, *CNS* central nervous system

### Exposure-efficacy analysis

In the first step of the analysis, the base model which included the effect of exposure as the log(*C*_trough,ss_) was associated with the lowest BIC value of all explored exposure functional forms. A model evaluating an *E*_max_-type exposure function resulted in a similar BIC; however, the estimated EC_50_ parameter did not appear biologically reasonable and was associated with large imprecision (standard-error). Therefore, the model with log(*C*_trough,ss_) was taken forward as the base model. During the univariate screening of covariates, log(baseline tumor size), number of days of prior crizotinib treatment duration, body weight (BW), body mass index (BMI), and body surface area (BSA) were all associated with a decrease in BIC (Supplemental Table 1). Given BW, BMI, and BSA are correlated and BW showed the greatest change in BIC, only BW was subsequently investigated in an expanded model. During the forward addition process, the addition of log(baseline tumor size) and prior crizotinib treatment duration reduced the BIC relative to the reference models while BW did not improve the model (i.e., did not further reduce the BIC). Finally, during backward elimination of covariates, the removal of alectinib exposure reduced the BIC in this reduced model when compared with full model. The final model included the effects of log(baseline tumor size) and prior crizotinib treatment duration. No statistically significant effect of alectinib exposure was identified in the final CPH model (95% CI for an exposure effect includes 1).

**Fig. 2:**
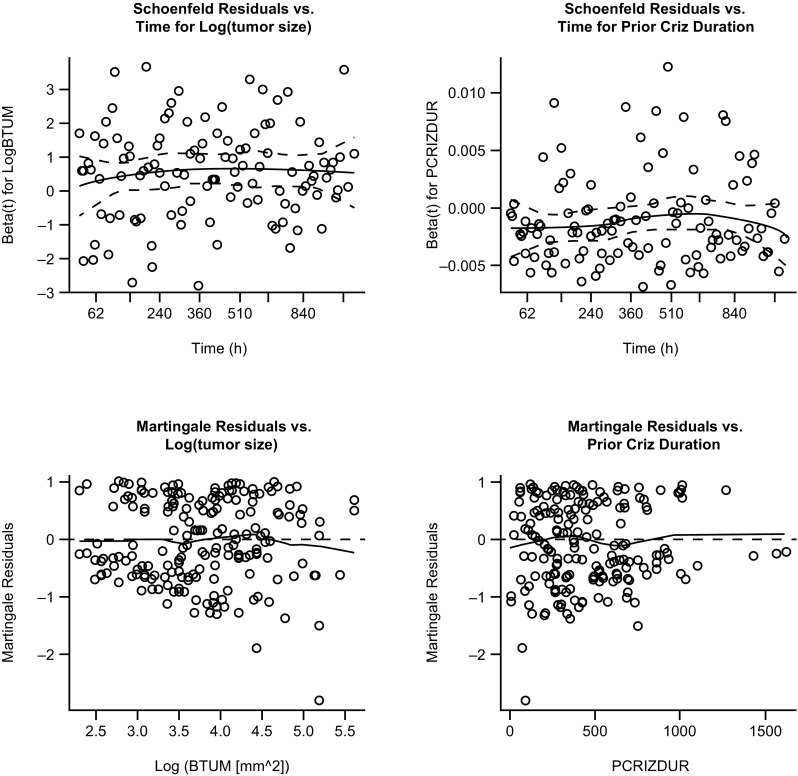
Diagnostic plots from the final Cox proportional hazards model. Top: Schoenfeld residuals from the final model. Solid lines represent locally smoothed fit to the Schoenfeld residuals, and the dash lines represent ± 2-standard-error around the fit. Bottom: Martingale residuals from the final model. The solid line represents locally smoothed fit to the martingale residuals. Martingale residuals should have a range between − ∞ and 1 and mean of zero, and should show no strong trends

**Fig. 3:**
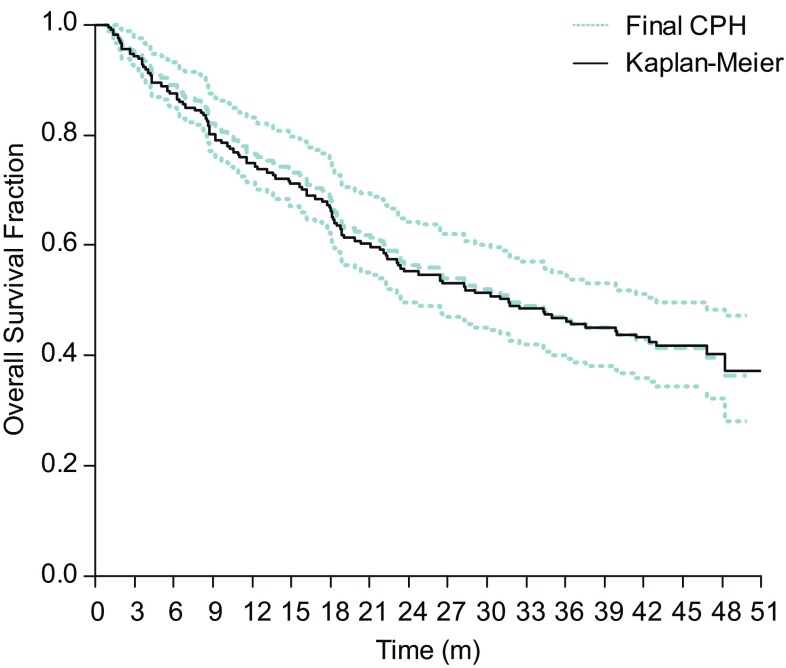
Overlay of the final Cox proportional hazards model (CPH) and associated 95% CIs and the nonparametric Kaplan–Meier analysis. The solid black line represents the observed OS from the nonparametric Kaplan–Meier analysis of the final pooled phase II OS data. The dashed lines represent the final CPH model estimate and associated 95% CIs

Diagnostic plots of Schoenfeld residuals for the final model revealed no systematic deviations from horizontal for the smoothed fit of the scatter over time supporting the assumption of proportional hazards for both identified covariates (Fig. 2). Plots of Martingale residuals against the identified covariates in the final model illustrated no evidence for nonlinearity with the smoothed lines close to horizontal (Fig. 2). The performance of the final model is illustrated in an overlay plot showing that the final CPH model and associated 95% CIs capture well the observed OS in the corresponding nonparametric Kaplan–Meier analysis (Fig. 3).

**Fig. 4:**
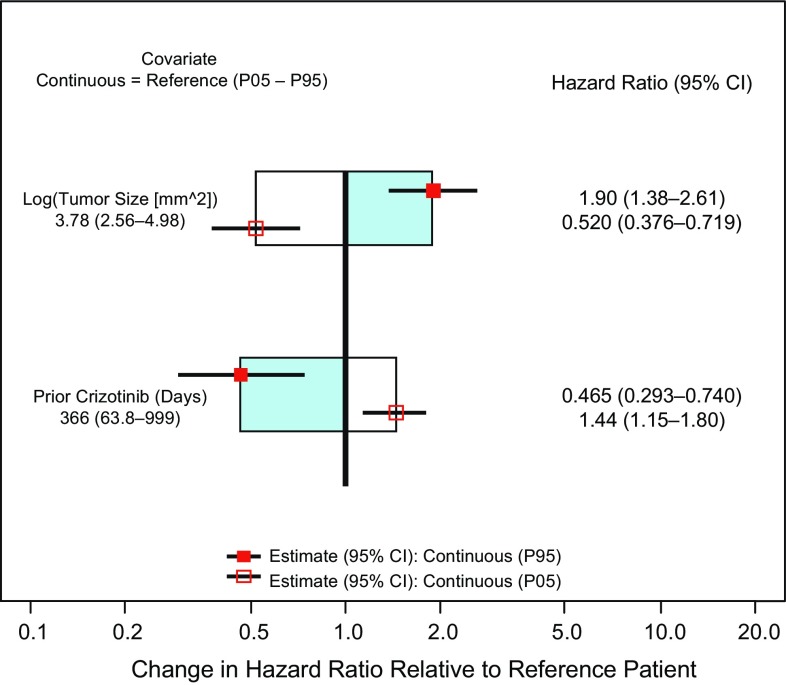
Covariates from the final Cox proportional hazards model

The covariates in the final model are illustrated in Fig. 4. Results provide an estimate HR (95% CI) for the 5th and 95th percentile of these continuous covariates relative to the median value. As seen in Fig. 4, larger baseline tumor size and shorter prior crizotinib treatment duration are associated with worse survival.

### Exposure-safety analysis

**Fig. 5:**
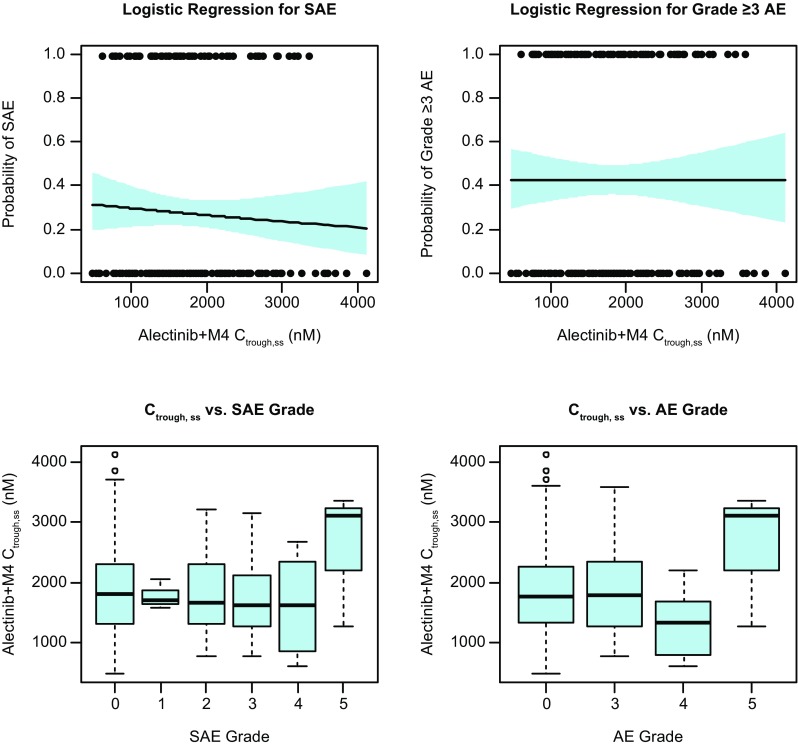
Exposure-safety analysis. Top: univariate logistic regression analysis for the assessment of the relationship between *C*_trough,ss_ and SAEs (left) or Grade ≥ 3 AEs (right). The black points represent the individual exposures for patients reporting (1.0) or not reporting (0.0) the safety event. The solid black line represents the logistic regression fit along with the 95% CIs (blue shading) for the fit; Bottom: Boxplot distributions of *C*_trough,ss_ by grades of SAEs (left) or Grade ≥ 3 AEs (right). Median values of *C*_trough,ss_ are designated by black lines in the center of the boxes. Boxes indicate the inter-quartile range (IQR). Whiskers represent 1.5*IQR. Outliers are marked outside of the whiskers by circles

Univariate logistic regression analyses for the relationship between alectinib exposure and incidence of SAEs or Grade ≥ 3 AEs revealed no apparent relationship across the nearly ninefold exposure range included in the analysis (Fig. 5). Statistical assessment supports no statistically significance for either SAEs or Grade ≥ 3 AE by Chi-square statistic (*P* = 0.465 and *P* = 0.978, respectively). The distribution of alectinib exposures by individual grades of SAEs or Grade ≥ 3 AEs further support no relationship between alectinib exposure and severity of safety events (*P* ≥ 0.597 by ANOVA) and illustrate substantial overlap in the distribution of exposure per safety event grade (Fig. 5). Of note, there was a low incidence (*n* = 3 [1.4%]) of Grade 5 safety events in the exposure–response population.

## Discussion

Alectinib is a novel, CNS-active ALK inhibitor, which has demonstrated robust efficacy and good safety in patients with *ALK*-positive NSCLC who have progressed on, or are intolerant to, crizotinib treatment. Updated data from a pooled analysis of the phase II studies (NP28673; global [NCT01801111] and NP28761; North American [NCT01871805]) demonstrated a median OS of greater than 2 years in patients with pretreated *ALK*-positive NSCLC receiving alectinib, and a good tolerability profile [13].

Dose selection and justification are key elements in the development of new agents and central to the demonstration of benefit/risk. However, within the field of oncology, the approaches to dose selection appear antiquated and rely frequently on methodology developed for cytotoxic chemotherapies with a goal of identifying a maximally tolerated dose (MTD) [19]. This assumes that the maximal effect is linked to achieving the maximum dose/exposure approaching early toxicity in a small number of patients but often does not consider long-term safety as well as efficacy outcomes in justifying the selected dose. This has resulted in a large number of post-marketing requirements and commitments for dose optimization investigations for many newly registered agents [16, 19]. With the goal of dose selection to maximize the expected efficacy benefit while minimizing the expected toxicity, it is critical to investigate the relationship between exposure and long-term outcomes for justifying the selected dose.

The present analysis investigated the relationship between alectinib exposure and the key efficacy and safety outcomes from the final pooled phase II data in the crizotinib-resistant *ALK*-positive NSCLC population. The investigation of exposure–efficacy relationships was through a multivariate CPH analysis which considered the potential modulatory effects of 5 functional forms of exposure along with 14 covariates in contributing to the survival of patients. The use of CPH model for survival analysis is well established and enables an identification and quantification of covariates influencing outcomes [29, 30]. Additionally, the present analyses investigated the relationship between alectinib exposure and key safety endpoints.

The results of the exposure–response analyses of the pooled phase II studies demonstrated no significant effect of alectinib exposure on OS following administration of the recommended alectinib 600 mg BID dose in the crizotinib-resistant *ALK*-positive NSCLC population. This analysis indicates that patients derive similar benefit from alectinib treatment across the wide nearly ninefold exposure range when accounting for the modulatory effects of the identified significant covariates. These results justify the recommended global alectinib 600 mg BID dose selection to provide systemic exposures of alectinib within the plateau range of response for OS. Population PK analysis of alectinib previously showed that the major covariate influencing alectinib and M4 PK was BW, with higher BW associated with lower exposure [32]. The lack of exposure–response relationship seen here supports that no dose adjustments would be needed by BW. In addition, the results support that no expected additional benefit in terms of improved efficacy is expected at dose/exposures exceeding the recommended alectinib 600 mg BID dose.

The analysis outcomes are complemented by a previously completed exploratory graphical pharmacokinetic/pharmacodynamic (PK/PD) analysis in the initial dose escalation, which showed that across the alectinib 300–900 mg BID dose range higher *C*_trough,ss_ was associated with higher reduction in tumor size and a plateau appeared to be reached at *C*_trough,ss_ corresponding to the 600 mg BID dose [32, 33]. That analysis suggested potential for reduced efficacy at doses/exposures lower than the 600 mg BID dose supporting it as the recommended alectinib global dose. Further, the primary analysis of the phase II studies showed that following alectinib 600 mg BID no significant relationship was seen between alectinib exposure and overall response rate (ORR) based on logistic regression analysis [11, 12, 32]. The present analysis builds on those initial observations by investigating the effect of exposure on long-term survival of patients. Of note, the previous exposure-ORR analysis utilized the population PK model predicted average concentration as the exposure measure. While that measure accounts for time on treatment and dose interruptions, the observed C_trough,ss_ used in this analysis provides a clinically relevant and practical exposure measure for assessment of exposure–response relationships. In any case, the two exposure measures are high correlated (*r*^2^ > 0.8) [Roche, data on file] supporting the use of either measure for analysis.

The results of this work contrast the outcomes seen from exposure–response analyses reported for the first generation ALK inhibitor, crizotinib [34–36]. While the recommended clinical dose for crizotinib was selected based on MTD, completed exposure–response analyses demonstrated a positive exposure–efficacy relationship suggesting that a proportion of patients receiving the approved crizotinib 250 mg BID dose may not be deriving full benefit from crizotinib therapy [34–36]. This resulted in an FDA mandated post-marketing commitment for further investigation of exposure–response relationships to evaluate the appropriateness of the approved 250 mg BID dose for all patients [34].

The final CPH analysis indicated that the major significant covariates impacting OS were the disease associated factors, baseline tumor size and prior crizotinib treatment duration. Baseline tumor size is frequently reported as a major covariate influencing the PK and/or PD of monoclonal antibodies as it contributes to target mediated drug disposition for antibodies targeting membrane bound antigens on tumor cells [37]. Given alectinib is a small molecule targeting the ALK kinase, the impact of baseline tumor size on OS from the alectinib phase II studies likely reflects the overall extent of disease burden in patients. Indeed, tumor size is an established prognostic factor for NSCLC with larger tumor size associated with poorer survival [38]. This was confirmed in an analysis of a large surveillance database containing more than 50,000 patients [39]. In this analysis, patients with a baseline tumor size representing the 95th percentile of the population were associated with a HR of 1.90 (95% CI: 1.38–2.61) relative to the median tumor size in the population. The other major covariate, prior crizotinib treatment duration, may be reflective of the overall health status of patients enrolled and/or their responsiveness to treatment. Patients within the 5th percentile of number of prior crizotinib treatment days were associated with a HR of 1.44 (95% CI: 1.15–1.80) relative to the median number of days. This may represent a poorer overall condition of some patients, which may not allow them to endure treatment. Alternatively, intratumoral heterogeneity could be contributing to treatment response for the targeted ALK inhibitors. In a prospective evaluation, an investigation of the evolution of NSCLC revealed widespread intratumor heterogeneity for both somatic copy-number alterations and mutations [40]. The intratumoral heterogeneity in NSCLC may contribute to resistance and/or progression after a period of treatment with targeted therapies, as only a portion of the heterogeneous tumor cells are destroyed on-treatment with the specific agent [41]. This could contribute to the shorter term responsiveness to crizotinib and subsequent shorter survival during alectinib treatment. Notwithstanding, the pooled analysis from the alectinib phase II studies demonstrated a clinically meaningful OS of greater than 2 years in patients already failing on crizotinib treatment, supporting its initial approval and use in this setting [13].

Interestingly, results from the analysis indicated that alectinib associated OS was not influenced by the presence of CNS metastases at baseline supporting benefit irrespective of this generally negative prognostic factor, albeit a relatively small sample size in the exposure–response population. These outcomes are supported by previously completed population PK analyses which demonstrated that alectinib PK is not influenced by presence of CNS metastases at baseline [32]. Conversely, it has been reported that nearly half of patients treated with crizotinib progress in the CNS, likely negatively impacting survival [42, 43]. Unlike crizotinib, alectinib is not a substrate of drug efflux transporters, p-glycoprotein (P-gp) or breast cancer resistance protein (BCRP), which are located at the blood–brain barrier and, therefore, may effectively penetrate and be retained in the CNS [9]. More recently, alectinib decreased the risk of CNS progression by 84% compared with crizotinib in head-to-head investigation in the first-line phase III trial, ALEX [14].

Results from the exposure–response analyses of the pooled alectinib phase II studies also demonstrated no significant relationship between alectinib exposure and incidence or severity of safety events following administration of the alectinib 600 mg BID dose. While the distribution of Grade 5 events appears visually higher compared with lower grade events, this is driven by the very few (*n* = 3) events and ANOVA testing confirms lack of statistical significance. The lack of exposure relationship to safety events across the nearly ninefold exposure range confirms the tolerability of the alectinib 600 mg BID dose. These outcomes are consistent with the initial exposure–response investigations during the primary analyses of the phase II studies [32], and are supportive of the overall well-tolerated safety profile for alectinib. Indeed, in the pooled analyses, only 14.7% of patients experienced AEs leading to dose reductions and 6.2% of patients experienced AEs leading to withdrawal over a median follow-up period of almost 2 years [13]. Conversely, completed exposure–response analyses for safety of another approved ALK inhibitor, ceritinib, showed a significant exposure-dependent effect on its toxicity [44, 45]. Given the poor tolerability and high incidence in dose reductions, an FDA mandated post-marketing requirement was issued for investigation of lower ceritinib doses to improve tolerability while maintaining its clinical benefit [44].

The present analysis has pooled two similarly designed phase II studies demonstrating similar results. Our analysis is, however, limited by the fact that the phase II studies were not designed to investigate exposure–response relationships and, therefore, this analysis is considered a post-hoc exploratory evaluation based on the data generated in the studies. In addition, while the completed CPH analysis investigated the potential effects of several functions of exposure and numerous covariates, there may still be unidentified risk factors influencing OS. Further, the CPH analysis may not entirely remove all confounding effects and does not account for potential interactions between covariates. Nonetheless, the favorable diagnostic plots and performance of the final CPH model evidenced in the visual assessment supports a robust final model in capturing OS. The lack of exposure-safety relationship across the wide exposure range supports the acceptable safety profile of alectinib.

## Conclusion

Results from the exposure–response analyses demonstrate that the alectinib 600 mg BID dosing regimen provides systemic exposures of alectinib within the plateau of response for OS while achieving exposures not associated with increased risk of toxicity and thus maintaining the good tolerability of alectinib. These analyses justify the recommended alectinib 600 mg BID dose for the global crizotinib-resistant *ALK*-positive NSCLC population. This dosing regimen has been approved in more than 50 countries to-date.

## Electronic supplementary material

Below is the link to the electronic supplementary material.

Supplementary material 1 (DOCX 15 KB)
