# Supplementary material for: Exposure–response analysis of alectinib in crizotinib-resistant ALK-positive non-small cell lung cancer
Source: Cancer Chemother Pharmacol. 2018 May 10;82(1):129–38. doi: 10.1007/s00280-018-3597-5 (PMC6010493; doi:10.1007/s00280-018-3597-5)
Supplement: Supplementary file 1 — Supplementary material 1 (DOCX 15 KB) [file 280_2018_3597_MOESM1_ESM.docx]

Article title: Exposure-response analysis of alectinib in crizotinib-resistant ALK-positive non-small cell lung cancer

Journal name: Cancer Chemotherapy and Pharmacology

Author names: Peter N. Morcos, Eveline Nueesch, Felix Jaminion, Elena Guerini, Joy C. Hsu, Walter Bordogna, Bogdana Balas, Francois Mercier

Affiliation and e-mail address of the corresponding author: Roche Innovation Center New York, NY, USA; email: [peter.morcos@roche.com](mailto:peter.morcos@roche.com)

**Supplemental Table 1.** Univariate covariate screening on the Base Model during Cox Proportional Hazards Model Building Process

| **Covariate** | **Coefficient** | **SE** | **RSE** | **HR** | **95% CI for HR** | **OF** | **BIC** |
| --- | --- | --- | --- | --- | --- | --- | --- |
| LogBTUM | -0.4975 | 0.2302 | 46.26 | 0.608 | 0.3872–0.9547 | 1065.5 | 1076.17 |
| PCRIZDUR | -0.558 | 0.2246 | 40.25 | 0.5723 | 0.3685–0.8889 | 1067.59 | 1078.26 |
| BTUM | -0.5487 | 0.2319 | 42.27 | 0.5777 | 0.3667–0.9102 | 1069.36 | 1080.03 |
| BW | -0.9103 | 0.2421 | 26.59 | 0.4024 | 0.2504–0.6467 | 1072.54 | 1083.21 |
| BMI | -0.7659 | 0.2312 | 30.19 | 0.4649 | 0.2955–0.7314 | 1072.78 | 1083.45 |
| BSA | -0.9367 | 0.2475 | 26.43 | 0.3919 | 0.2413–0.6367 | 1073.74 | 1084.41 |
| AGE | -0.6975 | 0.2201 | 31.56 | 0.4978 | 0.3234–0.7664 | 1075.42 | 1086.09 |
| ECOG | -0.5317 | 0.2277 | 42.82 | 0.5876 | 0.3761–0.9181 | 1075.48 | 1086.15 |
| RACE | -0.7247 | 0.2335 | 32.21 | 0.4844 | 0.3066–0.7655 | 1076.58 | 1092.58 |
| SMK | -0.6169 | 0.2308 | 37.42 | 0.5396 | 0.3432–0.8483 | 1077.86 | 1088.53 |
| CNSM | -0.7176 | 0.2285 | 31.84 | 0.4879 | 0.3118–0.7636 | 1078.28 | 1088.95 |
| CHEM | -0.6706 | 0.224 | 33.4 | 0.5114 | 0.3297–0.7932 | 1078.52 | 1089.19 |
| HT | -0.7697 | 0.2485 | 32.29 | 0.4631 | 0.2845–0.7538 | 1078.92 | 1089.59 |
| SEX | -0.694 | 0.232 | 33.43 | 0.4996 | 0.317–0.7872 | 1079.53 | 1090.2 |
| ETHN | -0.676 | 0.2248 | 33.26 | 0.5087 | 0.3274–0.7903 | 1079.55 | 1090.22 |

Note: OF and BIC values for the Base Model are 1079.63 and 1084.96, respectively.

SE: Standard error; RSE: residual standard error; HR: hazard ratio; CI: confidence interval; OF: objective function; BIC: bayesian Information criteria; LogBTUM: log of baseline tumor size; PCRIZDUR: prior crizotinib treatment duration; BW: body weight; BMI: body mass index; BSA: body surface area; SMK: smoking status (yes/no); CNSM: presence or absence of CNS metastases at baseline; CHEM: prior chemotherapy treatment (yes/no); HT: height; ETHN: ethnicity
